# Carboranes as unique pharmacophores in antitumor medicinal chemistry

**DOI:** 10.1016/j.omto.2022.01.005

**Published:** 2022-01-10

**Authors:** Yu Chen, Fukuan Du, Liyao Tang, Jinrun Xu, Yueshui Zhao, Xu Wu, Mingxing Li, Jing Shen, Qinglian Wen, Chi Hin Cho, Zhangang Xiao

**Affiliations:** 1 Laboratory of Molecular Pharmacology, Department of Pharmacology, School of Pharmacy, Southwest Medical University, Luzhou 646000, China; 2 Department of Oncology, Affiliated Hospital of Southwest Medical University, Luzhou 646000, China; 3 Luzhou Key Laboratory of Cell Therapy & Cell Drugs, Southwest Medical University, Luzhou 646000, China; 4 Faculty of Medicine, School of Biomedical Sciences, The Chinese University of Hong Kong, Hong Kong 999077, China

**Keywords:** carborane, cage, antitumor, pharmacophore, drug design

## Abstract

Carborane is a carbon-boron molecular cluster that can be viewed as a 3D analog of benzene. It features special physical and chemical properties, and thus has the potential to serve as a new type of pharmacophore for drug design and discovery. Based on the relative positions of two cage carbons, icosahedral *closo*-carboranes can be classified into three isomers, *ortho*-carborane (*o*-carborane, 1,2-C_2_B_10_H_12_), *meta*-carborane (*m*-carborane, 1,7-C_2_B_10_H_12_), and *para*-carborane (*p*-carborane, 1,12-C_2_B_10_H_12_), and all of them can be deboronated to generate their *nido*- forms. Cage compound carborane and its derivatives have been demonstrated as useful chemical entities in antitumor medicinal chemistry. The applications of carboranes and their derivatives in the field of antitumor research mainly include boron neutron capture therapy (BNCT), as BNCT/photodynamic therapy dual sensitizers, and as anticancer ligands. This review summarizes the research progress on carboranes achieved up to October 2021, with particular emphasis on signaling transduction pathways, chemical structures, and mechanistic considerations of using carboranes.

## Graphical abstract

**Figure:**
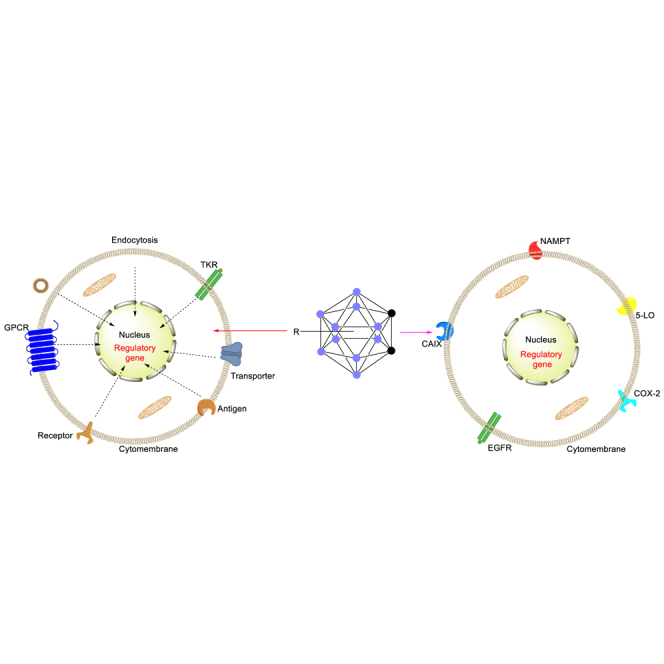

## Teaser

The applications of carboranes in the field of antitumor treatments mainly include BNCT, BNCT/PDT dual sensitizers, and anticancer ligands. This review summarizes the research progress on carboranes as useful pharmacophores in this field, with particular emphasis on signaling transduction pathways, chemical structures, and mechanistic considerations.

## Introduction

**Figure 1:**
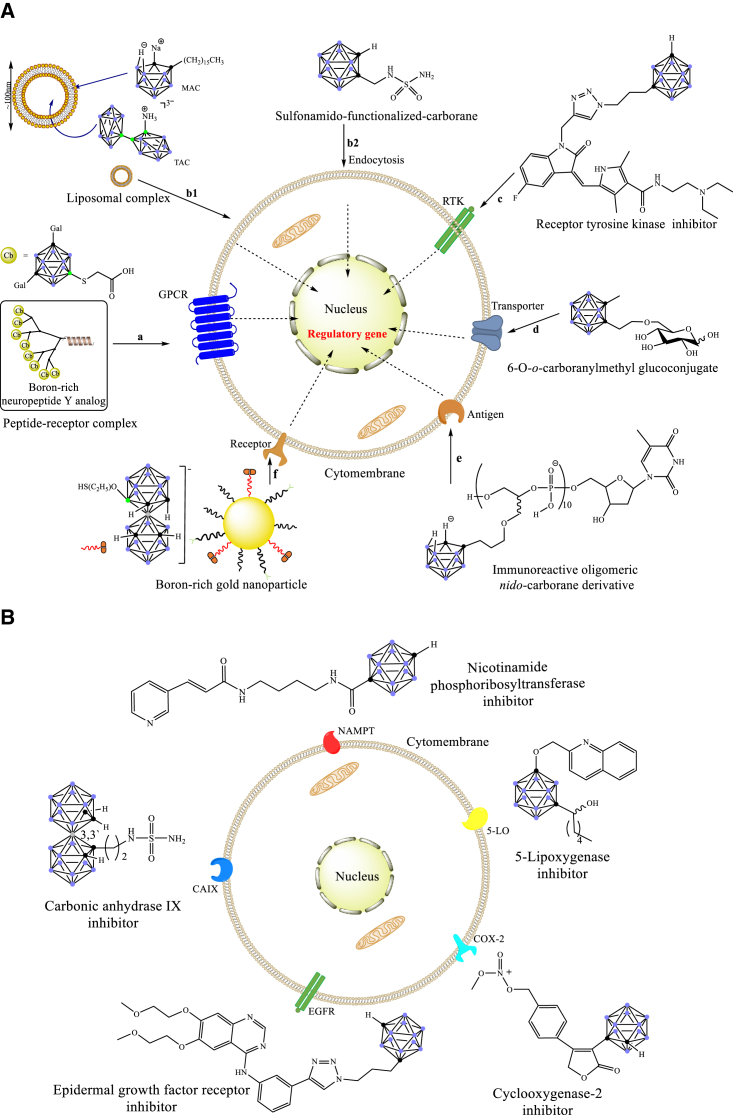
Interaction of carborane derivatives and cancer cells (A) Schematic representation of the routes of carborane derivatives entering cancer cells. (B) Carboranes bind to the skeleton of different enzyme inhibitors and interfere with receptors.

Carboranes, boron-carbon molecular cage compounds, are often viewed as the 3D analogs of benzene.^1^ They have a wide range of applications as useful functional building blocks in material science,^2^, ^3^, ^4^, ^5^, ^6^, ^7^, ^8^, ^9^, ^10^ organometallic/coordination chemistry,^11^, ^12^, ^13^, ^14^, ^15^, ^16^, ^17^ and medicinal chemistry.^18^, ^19^, ^20^, ^21^ In this context, considerable progress has been made in carborane functionalization.^22^, ^23^, ^24^, ^25^, ^26^, ^27^, ^28^ The special physical and chemical properties of carboranes allow the design of carborane-containing molecules with new and better antitumor activities, and thus offer medicinal chemists a unique opportunity to explore these new chemical entities for cancer therapy.^1^^,^^18^, ^19^, ^20^, ^21^ The most recent comprehensive review regarding carboranes as pharmacophores in medicinal chemistry, by Scholz and Hey-Hawkins, appeared a decade ago,^18^ which did not cover the recent research progress in this area. This review highlights the major achievements in the field of carboranes as pharmacophores in antitumor medicinal chemistry, with particular emphasis on signaling transduction pathways, chemical structures, and mechanistic considerations (Figure 1).

## Applications of carboranes as boron neutron capture therapy agents

**Figure 2:**
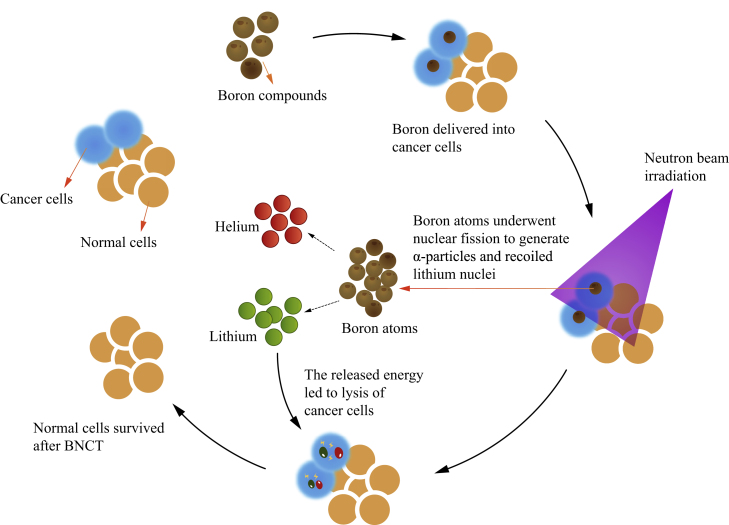
How BNCT kills tumor cells

**Figure 3:**
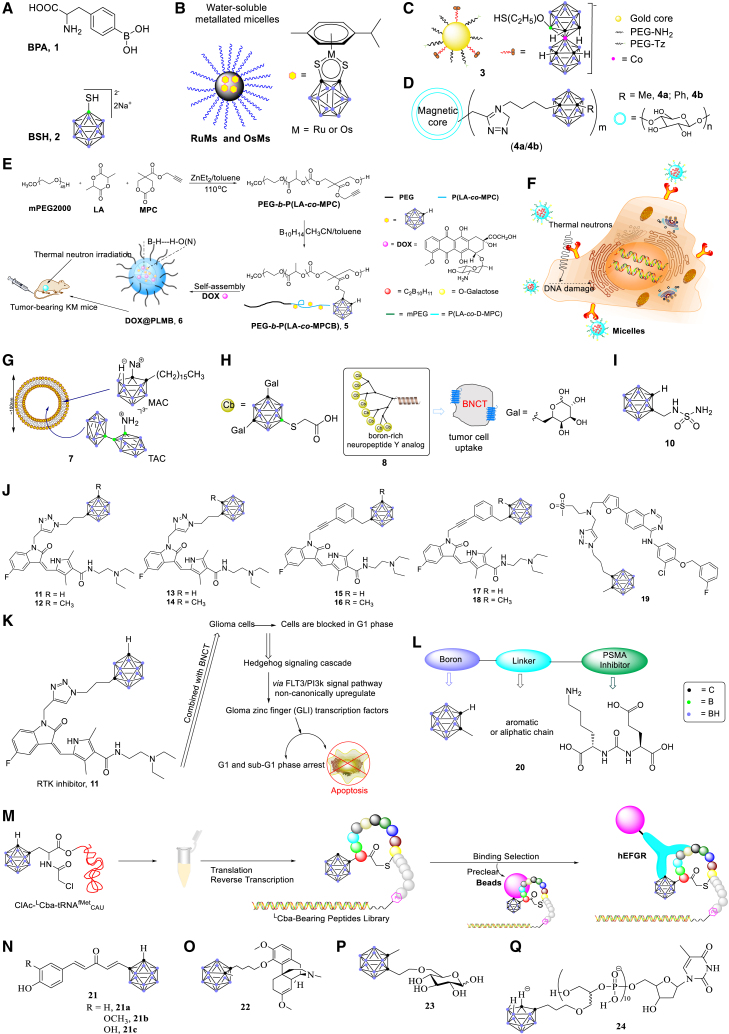
Applications of carboranes in BNCT

In boron neutron capture therapy (BNCT), the first step is the selective accumulation of ^10^B-containing compounds in cancer cells, which can be irradiated by low-energy and harmless thermal neutrons.^29^, ^30^, ^31^, ^32^, ^33^, ^34^, ^35^, ^36^ Subsequently, ^10^B atoms break up into α particles and lithium nuclei, yielding high linear energy transfer (LET) particles (Figure 2).^29^, ^30^, ^31^, ^32^, ^33^, ^34^, ^35^, ^36^ As a result, ^10^B-containing cancer cells can be destroyed by the high-LET particles. In contrast, the surrounding normal/healthy cells can survive because of the limited path length of these particles of only 5–9 μm, which is smaller than the diameter of a general cell.^29^^,^^35^ If ^10^B-containing compounds only accumulated in cancer cells, the thermal neutron irradiation would selectively eliminate tumors under BNCT conditions (Figure 2).^36^ Therefore, it is critical to selectively deliver large amounts of ^10^B-containing compounds into cancer cells rather than normal cells. However, only two boron-containing compounds, (L)-4-dihydroxy-borylphenylalanine (BPA, **1**) and sodium mercaptoundecahydro-*closo*-dodecarborate (BSH, **2**) (Figure 3A), are currently available as BNCT agents in clinical use.^37^ This may be ascribed to the low selectivity for cancer cells except for brain tumors as well as head and neck cancers (HNC).^38^

Carboranes possess unique physical and chemical properties. They have high content of ^10^B atoms and the highest neutron capture cross-section. Thus they can be ideal candidates for BNCT.^39^ Currently, a considerable number of carborane-containing compounds have been investigated for BNCT and are discussed in the following.

### Carboranes bound to nanoparticles

Nanoparticles are able to enhance permeability and retention effects as well as targeting effects, and thus can deliver ^10^B atoms into tumor tissues with high concentrations.^40^, ^41^, ^42^, ^43^, ^44^, ^45^ Using nanomaterials to deliver ^10^B-containing compounds for BNCT can potentially improve drug accumulation in tumor tissues.^46^ Hydrophobic carborane fragments and the polymerized nanoparticles can be formed on a single backbone chain.^47^ Carborane derivatives bound to nanoparticles have many advantages, including high stability, high accumulation in cancer cells, and ready preparation, and have become potential drug-delivery systems for nanomedicines and BNCT.^46^^,^^47^

Ruthenium and osmium are a class of transition metals widely used in cancer therapy.^48^, ^49^, ^50^, ^51^, ^52^, ^53^, ^54^, ^55^ Complexes containing electron-deficient ruthenium and osmium carborane were reported by the Sadler and Hanna groups.^50^^,^^51^ The redox-active response of these carborane-containing complexes to biomolecules have resulted in their potential application in cancer therapy.^48^ Additionally, Pluronic is a kind of amphiphilic block copolymer with good biocompatibility whose nanostructures have been widely used in biomedical fields.^52^, ^53^, ^54^, ^55^ Sadler and co-workers employed Pluronic triblock copolymer P123 micelles to encapsulate 16 electron complexes, yielding polymer micelles RuMs and OsMs in water through the self-assembly of nanoparticles (Figure 3B).^50^ They showed greater selectivity to cancer cells as well as higher intracellular boron concentrations compared with normal cells.^50^^,^^52^ These findings have provided promising complexes for BNCT.

Llop and co-workers recently reported that boron-rich gold nanoparticles (AuNPs, **3**) (Figure 3C) could serve as drug carriers for BNCT.^56^ Multifunctional AuNPs (core diameter 4.1 ± 1.5 nm) were synthesized as drug carriers with potential applications in BNCT.^56^ On the other hand, the Wang group reported that self-assembled gold nanoclusters were able to combine with carborane amino derivatives with good biocompatibility and stability.^57^ They achieved selective delivery of carborane derivatives into tumor tissues through the enhanced permeability and retention effect as well as a nanoscale effect.^57^

Among the nanoscale boron carriers, the carborane-loaded nanoscale covalent organic polymers (BCOPs) and magnetic nanoparticles are effective carriers in the BNCT.^58^, ^59^, ^60^, ^61^, ^62^ Multifunctional BCOPs were prepared from a Schiff base condensation reaction and further functionalized into BCOP-5T (five octyl chains) through alkyl chain engineering and size adjustment.^58^ With the loading of carborane, the 1,2-distearoyl-*sn*-glycero-3-phosphoethanolamine-N-[amino-(polyethyleneglycol)-2000] (M_w_ = 2000) coated with BCOP-5T exhibits excellent physiological stability and biocompatibility, and has been used as a carborane-loaded nanocarrier in BNCT.^58^ Hosmane and co-workers showed that through the click reaction, the carborane cage was successfully adsorbed into the modified magnetic nanoparticles (**4**) (Figure 3D).^61^

Doxorubicin (DOX) is an anthracycline antitumor antibiotic with a variety of hydroxyl and amino motifs.^63^, ^64^, ^65^, ^66^, ^67^, ^68^ Although DOX has been widely used in the treatment of a variety of cancers, serious cardiotoxicity is a major challenge.^63^ Nanoparticle delivery systems can potentially improve the efficacy and reduce the toxicity of DOX.^64^, ^65^, ^66^^,^^69^ The Yan group reported that DOX combined with nanoparticles reduced the toxicity in the treatment of liver cancer.^67^

To achieve combined administration of DOX in BNCT and chemotherapy (Figure 3E), carborane conjugated amphiphilic copolymer PEG-*b*-P (LA-*co*-MPCB) (PLMB, **5**) nanoparticles were synthesized by Huang and colleagues.^68^ DOX@PLMB (**6**) was formed by self-assembly of nanoparticles, which prevented boron compounds from leakage into the bloodstream by virtue of the covalent bond between carborane and the backbone chain of polymerization.^68^ Also, it was able to protect DOX from bursting release due to its dihydrogen bonding with carborane. Moreover, these authors found that the blood circulation time of DOX@PLMB was prolonged and boron accumulation was increased in tumor tissues. Therefore, DOX@PLMB has reduced systemic toxicity and an improved therapeutic effect.^68^

### Carboranes bound to PEG/liposomes

Hepatocellular carcinoma (HCC) is the most common cause of death from cancer.^70^, ^71^, ^72^, ^73^, ^74^, ^75^ D’Souza and Devarajan reported that the asialoglycoprotein receptor (ASGPR) could serve as an ideal target specific for delivery to HCC.^76^ The Zhou group reported that galactose and lactose residues had a strong affinity for ASGPR.^77^ The carborane-containing clusters self-assembled into micelles with HCC-targeting property were synthesized by Liu and co-workers (Figure 3F).^78^ Compared with BSH, the carborane-containing micelles enhance the selectivity and absorptive capacity of hepatoma carcinoma cells, and thus the cytotoxicity is reduced. The micelles can weaken the migratory behavior and induce apoptosis of cancer cells by destroying double-stranded DNA during cancer treatment.^78^

Among the macromolecular substances, liposomes fused with cell membranes delivered boron-containing elements into tumor tissues.^79^, ^80^, ^81^, ^82^, ^83^ Hawthorne’s group reported using liposomes as a transport medium for boron elements.^79^^,^^80^ Liposomes were converted into an ammonio derivative, Na_3_[1-(2′-B_10_H_9_)-2NH_3_B_10_H_8_] (TAC), which provided high concentration and long residence time of boron in cancer cells. Hawthorne and co-workers also designed a complementary lipophilic reagent, K[*nido*-7CH_3_(CH_2_)_15_-7,8-C_2_B_9_H_11_] (MAC), which could be stably incorporated into the liposomal bilayer (Figure 3G).^79^ Subsequently, the liposomal bilayer specifically bound to receptors on the surface of cancer cells and entered into cancer cells through endocytosis.^79^ It was demonstrated that the inclusion of amphoteric *nido*-carborane in the liposomal bilayer accumulated a high concentration of boron in tumor tissues.^79^ Also, the application of BNCT was able to encapsulate carboranes in small unilamellar liposomes to selectively deliver ^10^B to synovial tissues of patients with rheumatoid arthritis (RA).^84^ Moreover, as polyethylene glycol (PEG) is biocompatible,^85^, ^86^, ^87^ the carborane-PEG conjugate has been used as a new type of boron carrier.^85^, ^86^, ^87^ The simple encapsulation of *nido*-carborane anions in PEG liposomes as the boron carriers of BNCT was reported by Lee et al.^85^ PEGylated liposomes effectively delivered boron compounds by encapsulating carborane and delivering it into tumor cells.^85^

### Carboranes bound to peptide ligands

G-protein-coupled receptors (GPCRs) refer to a large family of cell-surface receptors.^88^, ^89^, ^90^, ^91^, ^92^ As a seven-pass transmembrane protein, GPCRs that are overexpressed on the membrane of cancer cells bound to peptide ligands and thus could be used as a shuttle for tumor-directed boron absorption systems (Figure 3H).^88^ Among them, human Y1 receptor (hY1R),^88^^,^^89^ gastrin-releasing peptide receptor (GRPR),^90^ and ghrelin receptor (GhrR)^91^^,^^92^ have become viable targets for BNCT by virtue of their high expression on the surface of cancer cells and their ability to internalize the bound ligands. In this context, the groups of Beck-Sickinger^88^ and Hey-Hawkins^89^, ^90^, ^91^ reported that the combination of carborane and hY1R, GRPR, or GhrR could represent a boron delivery agent in BNCT for the delivery of therapeutic drugs to cancer cells. Neuropeptide Y (NPY), a peptide of the three-membered NPY hormone family,^88^^,^^89^ bound to hY1R to form an NPY complex (**8**), and the complex was internalized into cells through receptor-mediated endocytosis. Carborane was introduced into the NPY complex by solid-phase peptide synthesis. A boron-modified NPY complex was then used as the boron carrier of BNCT, which selectively delivered therapeutic drugs into breast cancer cells.^88^^,^^89^ The significant overexpression of GRPR in various malignant tumor tissues makes it a very attractive target,^90^^,^^91^ whereby carborane could be attached to the peptide conjugates targeting tumor cells through GPCRs overexpressed on cancer cell membranes. Similarly, the expression of GhrR on a variety of cancer cells makes it a viable target for BNCT.^91^^,^^92^ GhrR could serve as a delivery system of BNCT to deliver high doses of boron into cancer cells.^92^

### Carboranes bound to enzyme/receptor inhibitors

**Figure 4:**
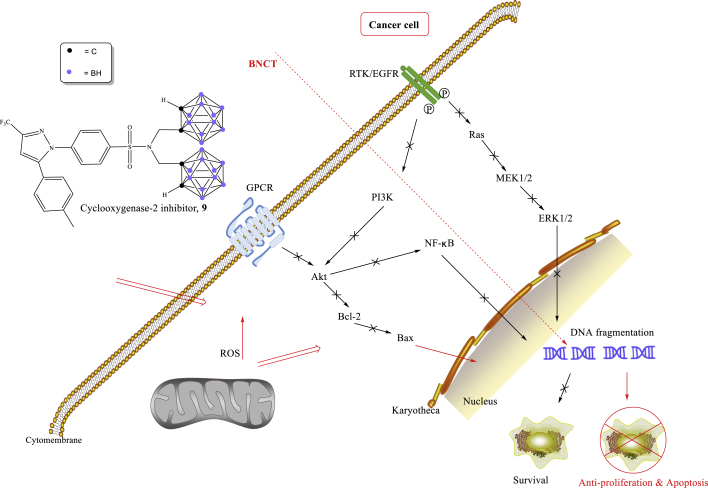
Signaling pathways of a carborane-derived COX-2 inhibitor

Cyclooxygenase-2 (COX-2) is highly expressed in HNC,^93^, ^94^, ^95^ which can be used as a potential target for HNC.^96^, ^97^, ^98^, ^99^ The Chen group developed a novel carborane-containing COX-2 inhibitor (**9**), which was able to induce apoptosis of cancer cells through BNCT and was effectively used for the treatment of HNC.^93^ It was shown that COX-2 inhibitor causes DNA double-strand breaks and forms reactive oxygen species, followed by downregulation of expression of phosphatidylinositol 3-kinase/protein kinase B (PI3K/Akt) and mitogen-activated protein kinase, finally inducing apoptosis of cancer cells in BNCT (Figure 4).^93^

Matrix metalloproteinases (MMPs) are a type of zinc-dependent endopeptidase that participate in the remodeling and degradation of all components of the extracellular matrix.^100^, ^101^, ^102^, ^103^, ^104^ It has been reported that carborane can combine with MMP ligands to deliver boron atoms into cancer cells, achieving the use of BNCT for dual therapy of tumors.^104^

Carbonic anhydrase IX (CAIX, **10**) is an enzyme overexpressed in mesothelioma and breast cancer cells.^105^, ^106^, ^107^, ^108^ CAIX inhibitors specifically bind to the low-density lipoprotein (LDL) receptor on the surface of cancer cells to form receptor-LDL complexes, and is delivered into cancer cells through endocytosis (Figure 3I).^105^ Sulfonamido-functionalized-carborane (CA-SF) was discovered by the Geninatti-Crich group.^105^ CA-SF served as a CAIX inhibitor and boron delivery agent and has been used in BNCT (Figure 3I) to inhibit the growth of mesothelioma and breast cancer cells.

Receptor tyrosine kinases (RTKs) are a subclass of tyrosine kinases that are involved in mediating cell-to-cell communication and controlling a wide range of complex biological functions.^109^, ^110^, ^111^, ^112^ Carborane-containing RTK inhibitors (**11**–**19**) were designed for the treatment of glioblastoma and prostate cancer (Figure 3J).^113^, ^114^, ^115^, ^116^ Recently, the combination of RTK inhibitors and BNCT, i.e., combination therapy, was suggested by Cerecetto and co-workers.^113^ They proposed a plausible mechanism whereby Hedgehog signal cascade non-canonically upregulated glioma zinc finger transcription factors via the FLT3/PI3K signaling pathway. RTK inhibitors specifically bound to receptors on the surface of cancer cells and were delivered into cancer cells through endocytosis (Figure 3K).^113^

Prostate-specific membrane antigen (PSMA), i.e., glutamate carboxypeptidase II, is an enzyme highly expressed on the surface of prostate cancer cells. PSMA is commonly used as a target for prostate cancer imaging and drug delivery.^117^, ^118^, ^119^, ^120^, ^121^ As boron-containing inhibitors generally had high binding affinity to PSMA, Flavell and co-workers combined PSMA inhibitor scaffolds with boric acids/carborane derivatives, delivering boron into prostate cancer cells and prostate tumor xenograft models (**20**, Figure 3L).^119^ The results showed that it was feasible to treat low-metastatic prostate cancer with PSMA containing boric acids or carboranes, thus demonstrating the potential role of PSMA in BNCT for the treatment of prostate cancer.^119^

Epidermal growth factor receptor (EGFR), a member of the transmembrane RTK family, is involved in promoting growth, proliferation, migration, angiogenesis, and chemotherapy resistance in tumors.^113^^,^^122^, ^123^, ^124^, ^125^ Antisense oligonucleotides conjugated with boron clusters (B-ASOs) could serve as potential gene expression inhibitors and boron carriers for BNCT,^126^, ^127^, ^128^, ^129^ providing a dual-action therapeutic platform. Some B-ASOs were designed to inhibit the biosynthesis of the EGFR for BNCT.^126^, ^127^, ^128^ Another example of combining carborane with EGFR was to integrate L-carboranylalanine (^L^Cba), an artificial cluster-type amino acid, into a peptide (Figure 3M), by which Suga and co-workers established a macrocyclic peptide library containing ^L^Cba residues.^130^ In this case, macrocyclic peptides have the advantages of high affinity for human EGFR (hEGFR) and high selectivity for hEGFR-expressing cells, as well as ready synthesis.

Curcumin, which is naturally present in turmeric plants, has been utilized to treat a variety of cancers and prevent Alzheimer's disease.^131^, ^132^, ^133^, ^134^, ^135^ Deagostino and co-workers found a new type of boronated analog of curcumin (**21**) that could be used in combination with BNCT.^132^ In this carborane-derived compound, β-diketone functionality was replaced by a carbonyl group while two phenolic rings were replaced by an *o*-carboranyl cage (Figure 3N).

### Carboranes bound to sinomenine

Sinomenine is a natural bioactive alkali extracted from the root of climbing ivy and has been widely used to relieve the symptoms of RA.^136^ Recently, Zhu and co-workers designed and synthesized^137^ a carborane-containing sinomenine derivative from sinomenine to treat RA (Figure 3O).^137^, ^138^, ^139^ They found that the uptake of boron and compound **22** in rat C6 glioma cells was significantly higher than that of BPA and BSH. Moreover, the concentration of boron in the cancer cells indicated that compound **22** had a higher permeability to the cell membrane, which was consistent with the results of the effectiveness of killing cancer cells *in vitro*.^137^

### Carboranes bound to carbohydrates/antibodies

Monosaccharides have been proven as another type of ideal candidate for BNCT, mainly due to their high water solubility, high biocompatibility, and low systemic toxicity.^140^, ^141^, ^142^, ^143^ The Ekholm group reported that monosaccharides could bind to carbohydrate transporters such as glucose transporters, and a glucoconjugate bearing an *o*-carboranylmethyl substituent (**23**) was designed and synthesized (Figure 3P).^142^ In addition, immune protein was proposed as a general boron delivery agent.^144^, ^145^, ^146^, ^147^ The bispecific antibody (BsMAb, **24**, Figure 3Q) was used to target tumor tissues by virtue of the tumor antigen specificity of BsMAb.^144^ In this context, BsMAb was discovered by Hawthorne and co-workers, providing an alternative method for site-directed boron targeting.^144^ This bispecific antibody can potentially be used as a boron delivery agent of BNCT for cancer treatment.

## Applications of carboranes as BNCT/photodynamic therapy dual sensitizer

Photodynamic therapy (PDT) and BNCT are promising cancer treatment modalities.^148^, ^149^, ^150^, ^151^, ^152^ Both approaches are based on the selective accumulation and retention of non-toxic sensitizer molecules (light or neutron sensitizers) in the target cells, whereby the target cells are treated by external radiation to activate the sensitizer, destroying the target cells.^148^ Dual therapies could thus improve therapeutic effectiveness by targeting different cellular components.^148^ Therefore, the synthesis of drugs with PDT and BNCT dual sensitizers have attracted much research interest.^153^, ^154^, ^155^

### Conjugates of chlorin e_6_ with iron bis(dicarbollide) nanoclusters

Chlorins can accumulate in tumor tissues and have been widely used as photosensitizers for PDT.^156^, ^157^, ^158^, ^159^ Moreover, chlorins have been utilized in conjugated boron nanoclusters, such as cobalt bis(dicarbonides), which were particularly attractive as boron-containing partial conjugates of chlorins.^160^ Semioshkin et al. and Viñas and co-workers demonstrated that cobalt bis(dicarbollides) were non-toxic both *in vivo* and *in vitro*.^161^^,^^162^

**Figure 5:**
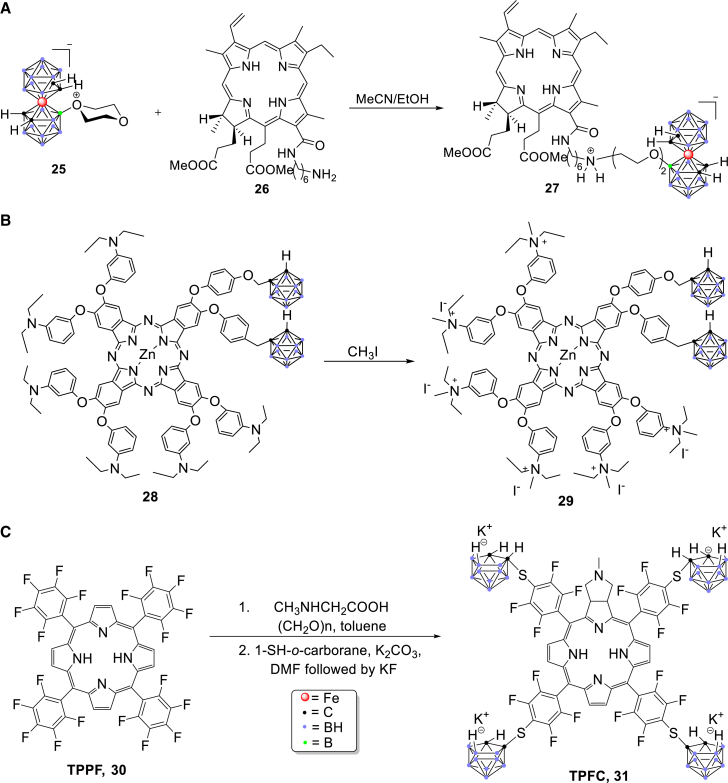
Synthesis of carborane-derived PDT/BNCT dual sensitizers

Conjugate of chlorin *e*_6_ with iron bis(dicarbollide) nanocluster (**27**), a dual sensitizer of BNCT and PDT, was synthesized by the Feofanov group (Figure 5A).^163^ They found that conjugate **27** accumulated effectively in rat glioblastoma, delivering >10^9^ boron atoms per C6 cell (rat glioblastoma C6 cell), which resulted in 50% and 90% of photoinduced cell death with the concentrations of 35 ± 3 and 80 ± 3 nM, respectively. Therefore, this conjugate provided an alternative direction for further research regarding the combination of PDT and BNCT.^163^

### Phthalocyanine-*ortho*-carborane conjugates

Boronated tetrapyrrole derivatives are promising dual sensitizers for BNCT and PDT by virtue of their low cytotoxicity under dark conditions, high boron content, and good tumor affinity.^164^ Among these compounds, phthalocyanine (PC) has attracted much interest in cancer treatment due to its high singlet oxygen production capacity, high molar extinction coefficient, high optical stability, and strong near-infrared absorption capacity.^165^ In this context, a water-soluble, *o*-carborane-derived PC complex (**29**) was designed and obtained by Hamuryudan and co-workers.^166^ Carboranes served as the boron source of BNCT while PC played a role in PDT activation (Figure 5B). This combination greatly enhances tumor killing efficiency.

### Tetrakis(*p*-carboranylthio-tetrafluorophenyl)chlorins

Boronated porphyrins and their derivatives can preferentially accumulate in cancer cells with low dark cytotoxicity,^167^, ^168^, ^169^ and thus have potential applications as BNCT/PDT dual sensitizers.^167^, ^168^, ^169^, ^170^ The Vicente group reported that boronated chlorin killed T98G cells of human glioma,^171^ while Pandey and co-workers found that fluorinated substituents promoted photosensitivity.^172^ It was also found that fluorinated porphyrins had higher photokinetic activity than their non-fluorinated counterparts.^171^^,^^172^ Tetrafluorophenyl porphyrin (TPPF, **30**) was synthesized by Drain and co-workers through microwave reaction.^153^ Vicente and co-workers synthesized a novel carborane-derived sensitizer, tetrakis(*p*-carboranylthio-tetrafluorophenyl)chlorin (TPFC, **31**) from TPPF (Figure 5C).^154^ The same group also reported that F98 rat glioma cells and F98 rat glioma brain tumor model could be used to evaluate the applicability of TPFC as a sensitizer. In *in vitro* studies, TPFC was located close to the nuclei and was highly photosensitive.^173^ According to the results from Vicente and colleagues, the efficacy of TPFC in the treatment of F98 rat glioma was comparable with that of BPA.^154^^,^^173^ Therefore, TPFC is potentially a promising dual sensitizer for PDT and BNCT.

## Applications of carboranes as anticancer ligands

### Estrogen receptor ligands

**Figure 6:**
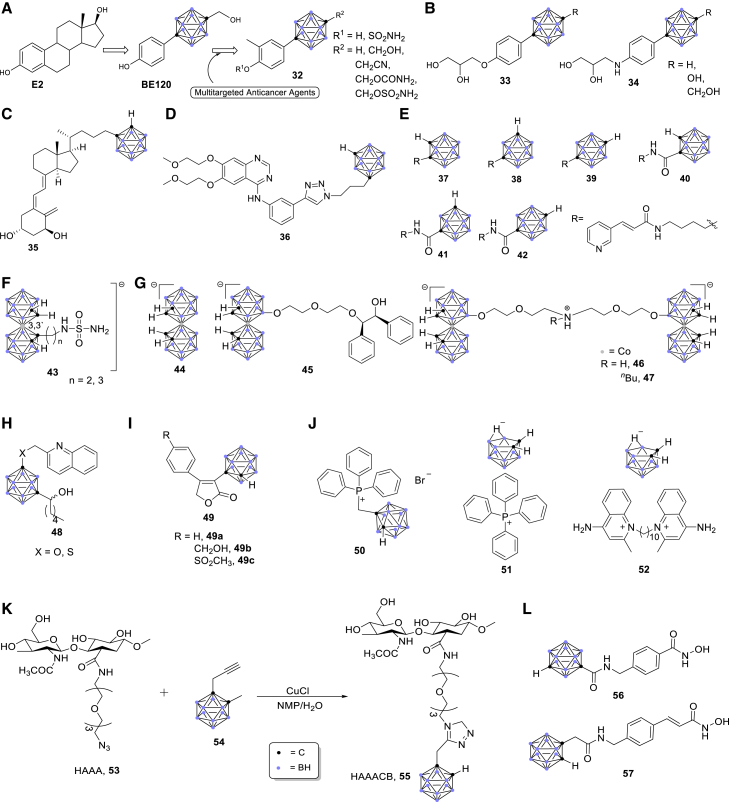
Applications of carboranes in anticancer ligands

Estrogen has important functions in cardiovascular, reproductive, skeletal, and central nervous systems.^174^, ^175^, ^176^, ^177^, ^178^, ^179^, ^180^, ^181^ E2 (17β-estradiol) and E1 (precursor of E2) were synthesized from estrone sulfate by steroid sulfatase (STS).^174^ Therefore, STS could be considered as a promising target for the treatment of breast cancer. In this context, Ohta and co-workers employed carborane as a hydrophobic pharmacophore, and several carborane-containing compounds were synthesized for the treatment of breast cancer.^174^^,^^182^ Among these, 1-(4-hydroxyphenyl)-12-hydroxymethyl-*p*-carborane (BE120) showed a potent binding ability to the estrogen receptor α (Figure 6A).^182^

### Androgen receptor ligands

**Figure 7:**
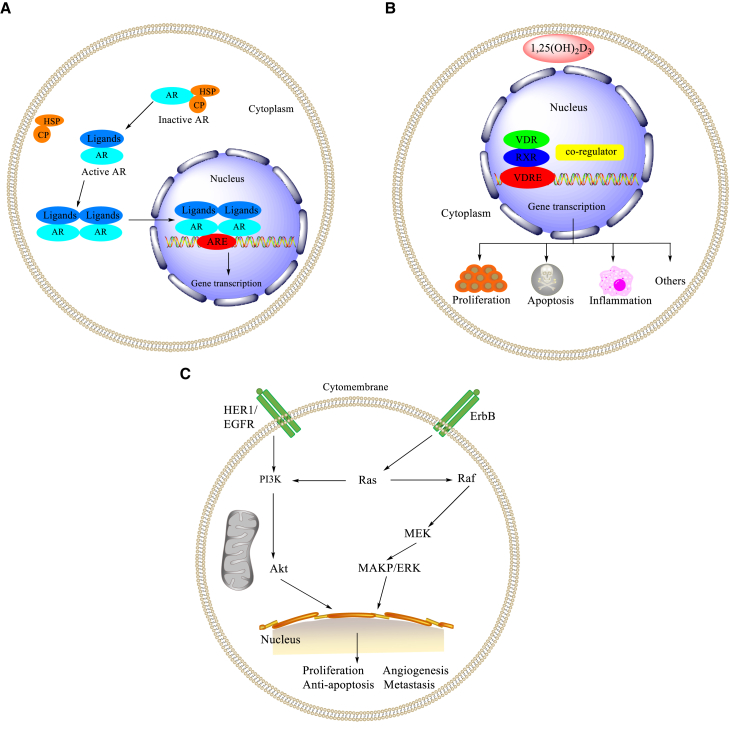
Mechanisms and signaling pathways (A) Mechanisms of gene regulation by AR. (B) Mechanisms of gene regulation by VDR. (C) Signaling pathways regulated by EGFR.

Similarly, the Ohta/Endo group developed carborane-derived compounds to target androgen receptor (AR).^183^^,^^184^ The AR homodimer translocated to nucleus and bound to the androgen response elements (AREs) in DNA, after which the AR-ARE complexes interacted with the promoters to regulate the target genes (Figure 7A).^185^ Subsequently, several carborane-containing AR ligands were developed as candidates for prostate cancer therapeutics.^186^, ^187^, ^188^ The carborane cage served as a hydrophobic pharmacophore for the AR ligand binding domain (AR LBD) of antagonists (**33**, **34**) (Figure 6B).^183^^,^^184^

### Vitamin D receptor ligands

Vitamin D nuclear receptor (VDR) is expressed in a variety of tumors and can be used as a potential target for cancer treatment.^189^, ^190^, ^191^, ^192^ A potent VDR agonist (**35**) with the combination of 1α,25-dihydroxyvitamin D3 (1,25D) and a carborane motif was designed and synthesized by Mouriño and colleagues,^193^ which represented the first example of vitamin D analog binding to the LBD of VDR (Figure 6C). 1,25D hormone regulates various physiological and pathological processes, such as cell proliferation and differentiation.^193^ In the nucleus, vitamin D and its analogs bind to VDR and then form the VDR-retinoid X receptor complexes, and bind to the vitamin D response element (Figure 7B).^189^ These results showed that carborane-derived vitamin D analogs could be employed for specific molecular recognition as well as anticancer drug design and discovery.

### Epidermal growth factor receptor ligands

EGFR/ErbB1 is a member of the ErbB protein family of RTKs.^194^, ^195^, ^196^, ^197^ The signaling pathways regulated by EGF-EGFR play key roles in regulating basic cell functions, such as cell proliferation, survival, differentiation, and migration (Figure 7C).^194^^,^^198^ EGFR-mediated cellular events are interfered with by inhibiting EGF from binding to EGFR on the surface of cancer cells.^199^, ^200^, ^201^ By employing the carborane cage as a pharmacophore, Viñas’ group demonstrated that a carborane-containing erlotinib derivative, 1,7-*closo*-carboranylanilinoquinazoline (**36**, Figure 6D), had a higher affinity than its parent compound erlotinib.^201^

### Nicotinamide phosphoribosyltransferase receptor ligands

Nicotinamide phosphoribosyltransferase (NAMPT) is a first rate-limiting enzyme in the cycle of mammalian nicotinamide adenine dinucleotide (NAD^+^).^202^, ^203^, ^204^, ^205^ Recent studies showed that NAMPT plays an essential role in metabolism, cell proliferation/survival, and inflammation.^202^, ^203^, ^204^, ^205^ In this context, a series of carborane-containing NAMPT inhibitors (**37**–**42**) were designed and synthesized by Nakamura and co-workers (Figure 6E).^202^ Among these inhibitors, compounds **41** and **42** showed significant NAMPT inhibitory activity with IC_50_ values of 0.098 ± 0.008 and 0.057 ± 0.001 μM, respectively (Figure 6E).^202^

### Carbonic anhydrase ligands

CAIX is an enzyme expressed on the surface of hypoxic tumor cells.^206^, ^207^, ^208^, ^209^ This enzyme promoted the survival of tumor cells and could be a target for anticancer therapy.^206^^,^^207^ In this context, biscarborane-containing CAIX inhibitors (**43**) were designed and synthesized by the Grüner group (Figure 6F).^206^^,^^210^ These cobalt bis(dicarbollide) ions acted as highly potent and specific CAIX inhibitors both *in vitro* and *in vivo*. Mechanistically, the crystal structure of the cobaltacarborane inhibitor bound to the CAIX active site; therefore, the enzyme cavity was able to easily accommodate the cobalt bis(dicarbollide) cluster (Figure 6F).^206^^,^^210^

### HIV protease receptor ligands

Human immunodeficiency virus 1 (HIV-1) protease is a potent target in the treatment of HIV-1 infection,^211^, ^212^, ^213^ and is also a target for anti-HIV drug design.^214^, ^215^, ^216^ The Konvalinka group discovered a series of novel non-peptide protease inhibitors that were able to inhibit a variety of protease inhibitor-resistant protease species (**44**–**47**, Figure 6G).^217^, ^218^, ^219^ These substituted metallacarboranes were effective and selective inhibitors of wild-type and mutated HIV proteases.

### 5-Lipoxygenase receptor ligands

5-Lipoxygenase (5-LO) acts as a catalyst for the conversion of arachidonic acid to leukotrienes.^220^, ^221^, ^222^, ^223^ The activity of 5-LO is affected by 5-lipoxygenase activating protein (FLAP), and Rev-5901 is an early inhibitor of FLAP-mediated 5-LO activation.^224^ As the introduction of carborane can improve the pharmacokinetic behavior of metabolically unstable drugs,^224^^,^^225^ the Hey-Hawkins group introduced carborane as a highly metabolically stable pharmacophore to the traditional 5-LO inhibitors (Figure 6H).^224^^,^^225^ Carborane-containing Rev-5901 derivatives showed the isosteric replacement of the phenyl ring by a carborane cage, leading to improved cytotoxicity in melanoma and colon cancer cells.^224^

### Cyclooxygenase ligands

COX-2 is involved in carcinogenesis, and increasing studies have been conducted on the potential cytotoxic properties of COX-2 selective inhibitors.^226^, ^227^, ^228^, ^229^ The incorporation of carborane units into the established anti-inflammatory drugs improved their metabolic stability.^226^ Hey-Hawkins and co-workers designed and synthesized several carborane-containing rofecoxib derivatives (**49**, Figure 6I), and these compounds showed superior selectivity against melanoma and colon cancer cells in comparison with normal cells.^226^

### Delocalized lipophilic cation ligands

The discovery of delocalized lipophilic cations (DLCs) is a milestone of organelle-specific drug delivery.^230^, ^231^, ^232^, ^233^ Owing to the high selectivity of growth arrest of DLC-functionalized carboranes for cancer cells, such as primary glioblastoma cancer stem cells, Vizirianakis and colleagues reported that DLC-functionalized carboranes (**50–52**) had potential applications in selective anticancer therapeutics (Figure 6J).^230^, ^231^, ^232^, ^233^ They also demonstrated that the target-specific DLC-functionalized carboranes could act as BNCT agents.^230^, ^231^, ^232^, ^233^

### Hyaluronic acid ligands

Hyaluronic acid is a highly biocompatible polysaccharide that plays an important role in cancer metastasis.^234^, ^235^, ^236^, ^237^, ^238^, ^239^, ^240^, ^241^, ^242^ It also interacts with various types of receptors that are overexpressed in cancer tissues.^234^, ^235^, ^236^ Introduction of carborane motifs can enhance the hydrophobic interaction between the bioactive compounds and their receptors and thus improve their stability and bioavailability *in vivo*.^237^, ^238^, ^239^, ^240^, ^241^, ^242^ Crescenzi and co-workers synthesized hyaluronan-amidoazido-carborane (HAAACB) (**55**) through a click-type coupling of hyaluronan-amidoazide and carboranyl alkyne (Figure 6K).^237^^,^^238^ They found that HAAACB could specifically interact with the CD44 receptor, leading to accumulation of boron atoms in cancer cells, which will have potential application in BNCT.^237^^,^^238^

### Histone deacetylase ligands

Histone deacetylases (HDACs) are known to be responsible for the global silencing of tumor-suppressor genes.^243^, ^244^, ^245^, ^246^, ^247^, ^248^, ^249^ Overexpression of HDACs was shown to be linked to several cancer types, and their selective inhibition results in potentiated anticancer effects.^249^ Treatment with histone deacetylase inhibitors (HDACis) can reverse this process and restore normal cell function. Therefore, HDACis have emerged as valuable epigenetic modulators for the treatment of cancers.^243^^,^^249^ Hansen and co-workers identified the *meta*-carboranyl hydroxamate **56** as the hit compound with an IC_50_ value of 0.006 μM and a more than 280-fold selectivity for HDAC6.^249^ To investigate the influence of the carborane moiety, they synthesized aryl analogs for the best pan-inhibitory compound **57** and the most selective HDAC6 inhibitor **56** (Figure 6L).^249^ Both **56** and **57** demonstrated synergistic anticancer activity when combined with the proteasome inhibitor bortezomib.^249^

## Conclusion

This review summarizes the recent advances in carborane-containing compounds with potential applications in antitumor treatments. Carboranes have been proven as useful pharmacophores in boron delivery agents for BNCT as well as hydrophobic drug carriers for certain biological targets. The introduction of carborane moieties into the skeletons of traditional organic compounds (hits, leads, drugs) can change the activity of the drugs or drug candidates and thus regulate their effects on cancer cells, as shown in the various examples discussed in this review. Although this research area is still in its infancy, selective functionalization of carboranes to obtain various carborane-containing derivatives has received increasing research attention. Application of carboranes as antitumor pharmacophores will open a new and specialized avenue for novel drug design and discovery.
